# A Systematic Review Establishing the Current State-of-the-Art, the Limitations, and the DESIRED Checklist in Studies of Direct Neural Interfacing With Robotic Gait Devices in Stroke Rehabilitation

**DOI:** 10.3389/fnins.2020.00578

**Published:** 2020-06-30

**Authors:** Olive Lennon, Michele Tonellato, Alessandra Del Felice, Roberto Di Marco, Caitriona Fingleton, Attila Korik, Eleonora Guanziroli, Franco Molteni, Christoph Guger, Rupert Otner, Damien Coyle

**Affiliations:** ^1^School of Public Health, Physiotherapy and Sports Science, University College Dublin, Dublin, Ireland; ^2^Department of Neuroscience, Rehabilitation Unit, University of Padova, Padova, Italy; ^3^Department of Neuroscience, NEUROMOVE-Rehab Laboratory, University of Padova, Padova, Italy; ^4^Padova Neuroscience Center, University of Padova, Padova, Italy; ^5^Department of Physiotherapy, Mater Misericordiae University Hospital, Dublin, Ireland; ^6^Intelligent Systems Research Centre, School of Computing, Engineering and Intelligent Systems, Ulster University, Derry, United Kingdom; ^7^Villa Beretta Rehabilitation Center, Valduce Hospital, Costa Masnaga, Italy; ^8^g.tec Medical Engineering GmbH, Schiedlberg, Austria

**Keywords:** stroke rehabilitation, robot-assisted gait trainer, electromyography, electroencephalography, human–machine interface, brain–computer interface

## Abstract

**Background:** Stroke is a disease with a high associated disability burden. Robotic-assisted gait training offers an opportunity for the practice intensity levels associated with good functional walking outcomes in this population. Neural interfacing technology, electroencephalography (EEG), or electromyography (EMG) can offer new strategies for robotic gait re-education after a stroke by promoting more active engagement in movement intent and/or neurophysiological feedback.

**Objectives:** This study identifies the current state-of-the-art and the limitations in direct neural interfacing with robotic gait devices in stroke rehabilitation.

**Methods:** A pre-registered systematic review was conducted using standardized search operators that included the presence of stroke and robotic gait training and neural biosignals (EMG and/or EEG) and was not limited by study type.

**Results:** From a total of 8,899 papers identified, 13 articles were considered for the final selection. Only five of the 13 studies received a strong or moderate quality rating as a clinical study. Three studies recorded EEG activity during robotic gait, two of which used EEG for BCI purposes. While demonstrating utility for decoding kinematic and EMG-related gait data, no EEG study has been identified to close the loop between robot and human. Twelve of the studies recorded EMG activity during or after robotic walking, primarily as an outcome measure. One study used multisource information fusion from EMG, joint angle, and force to modify robotic commands in real time, with higher error rates observed during active movement. A novel study identified used EMG data during robotic gait to derive the optimal, individualized robot-driven step trajectory.

**Conclusions:** Wide heterogeneity in the reporting and the purpose of neurobiosignal use during robotic gait training after a stroke exists. Neural interfacing with robotic gait after a stroke demonstrates promise as a future field of study. However, as a nascent area, direct neural interfacing with robotic gait after a stroke would benefit from a more standardized protocol for biosignal collection and processing and for robotic deployment. Appropriate reporting for clinical studies of this nature is also required with respect to the study type and the participants' characteristics.

## Introduction

Stroke, a disease with substantial personal and societal consequences, remains the leading cause of acquired disability worldwide. With 13.7 million new cases each year, the associated economic costs of treatment and post-stroke care are significant (Wilkins et al., 2017; Johnson et al., 2019). At 3 months after a stroke, 20% of people remain wheelchair dependent and ~70% walk with a reduced capacity (Mehrholz et al., 2017). Task-specific training is critical for recovery, and the intensity of practice is strongly associated with improved functional gait outcomes (Kwakkel et al., 2004; Veerbeek et al., 2014).

Providing high intensity restorative exercises for a larger share of the stroke population is part of the Action Plan for Stroke in Europe 2018–2030 (Norrving et al., 2018), yet the delivery of an adequate dosage of gait training for physically dependent patients is challenging in the rehabilitation sector, from manual handling and human resource perspectives. Robotic gait devices, which enable people to walk with electromechanical assistance to achieve a healthy gait trajectory, can potentially overcome some of these practical difficulties (Mehrholz et al., 2017; Cervera et al., 2018) and allow an intensive, high repetition of the gait cycle with reduced therapist involvement (as they no longer need to set the paretic limbs or assist trunk movements) (Sarasola-Sanz et al., 2017). The addition of robotic-assisted gait training (RAGT) to usual rehabilitation has been shown by a systematic review to improve the likelihood of regaining independent walking after a stroke [odds ratio 1.94, 95% confidence interval (CI), 1.39 to 2.71], with a subgroup analysis suggesting that people in the acute phase and non-ambulatory individuals benefit most from the intervention (Mehrholz et al., 2017). Of note is that the improvements in walking velocity and walking capacity did not match the observed improvements in independence in gait.

At present, RAGT alone has not been shown to be superior to equally dosed routine rehabilitation despite the increased intensity of stepping in RAGT (Taveggia et al., 2016; Bruni et al., 2018). When motor function is considered specifically as an outcome, the upper limb robotic devices have proven efficacy in contrast to the lower limb robotic training, where no treatment effect for motor function has been demonstrated (Lin et al., 2019). Current RAGT therapies have focused on providing high-intensity training and repetition but not on patient engagement, motivation, and reward, which are important factors for inducing cortical plasticity (Hogan et al., 2006; Goodman et al., 2014). Limitations in randomized controlled trials (RCTs) in this area to date have been identified (Molteni et al., 2018) and many RAGT protocols were criticized for allowing the trainee to be too passive, with lower metabolic costs, muscle activations, and subject effort reported in comparison to therapist-assisted treadmill training (Cai et al., 2006; Israel et al., 2006; Krishnan et al., 2013). However, rehabilitative robotics, when deployed correctly, have the ability to generate bottom up and top down complex and controlled multisensory stimulation to modify the plasticity of neural connections through the experience of movement (Molteni et al., 2018).

Direct human machine interfaces (HMIs) can translate electrical, magnetic, or metabolic activity at the brain or the muscle level into control signals for external devices (e.g., computers or neuroprosthetic and robotic devices) to replace, restore, or enhance the natural neural output (Wolpaw, 2012; Soekadar et al., 2015). Brain interfacing technology, primarily electroencephalography (EEG)-based brain computer interfaces (BCI,) can offer new strategies for robotic gait re-education after a stroke that can promote more active engagement in movement intent and/or by neurophysiological feedback. In stroke, BCI exploitation has mainly used motor imagery to drive brain activity levels (with no overt motor output) in combination with visual, auditory, or haptic feedback or to control an external device which executes the movement and provides proprioceptive feedback (Prasad et al., 2010; Van Dokkum et al., 2015). Of the nine upper limb studies identified in a recent systematic review of BCI for motor rehabilitation after a stroke, only three used BCI to control a robotic or orthotic device with large to moderate effect sizes noted for improved motor impairment (Cervera et al., 2018) and emerging evidence in upper limb rehabilitation now points to the superiority of BCI robotic training after a stroke to robotic training alone in motor recovery (Varkuti et al., 2013; Ang et al., 2014). No lower limb robotic RCT studies using BCI were reported in this review (Cervera et al., 2018).

Motor intent can also be determined non-invasively by electromyography (EMG) activity and responded to in triggered motion (Hussein et al., 2009) and thus has potential to enhance RAGT. EMG-based robotic movement has emerged as a well-developed field in upper limb rehabilitation in stroke (Ho et al., 2011; Vaca Benitez et al., 2013; Hu et al., 2015), and when used in robot-assisted rehabilitation has achieved a significantly higher completion rate compared to torque control for the participants with severe to moderate impairment in the upper limb (Paredes et al., 2015). EMG has also been combined with EEG in human–machine interactive force to improve the recognition of movement intent (Mrachacz-Kersting et al., 2012; Jiang et al., 2014; Bhagat et al., 2016).

As reported in a 2018 review of human intent-based control in motor rehabilitation after a stroke, most studies are in the laboratory stage (Li et al., 2018), and a systematic review of RCTs of BCI interfaces after a stroke identified no RAGT studies (Cervera et al., 2018). Therefore, the aim of this systematic review was to establish the current state-of-the-art in EMG and/or EEG neural biosignal deployment during robotic gait training post-stroke as described in the literature (with no limitation by study design applied). Contributing to this review is a panel of relevant stakeholders from the fields of rehabilitation, neurology, biomedical engineering, and BCI engineering who, in providing a summary of available data, comment and make important recommendations to standardize reporting and advance this important and emerging area in robotic-assisted gait rehabilitation in stroke.

The primary question that this review asks is:

what is the current state-of-the-art in neural–exoskeleton interface (non-invasive EEG and/or EMG) during robotic gait training after a stroke?

The secondary review questions asked are the following:

1. What is the profile of the stroke patients in the included studies?
2. What robotic gait devices are deployed?
3. What biosignals are measured in conjunction with the robotic gait devices and what devices (hardware and/or software) are used to capture these biosignals?
4. What protocols are used for recording and processing these biosignals?
5. For what purpose is the acquired biosignal data collected?

As a nascent area, the inclusive approach to study type was taken in this review to allow a true reflection of bioengineering translational research in gait rehabilitation robotics in a clinical population. A compendium of current data collection and signal processing procedures will be developed to allow recommendations for the standardization of future research in this field.

The systematic review was pre-registered with PROSPERO (PROSPERO 2018 CRD42018112252) (Heilinger et al., 2018).

## Methods

### Definitions

Prior to conducting the review, several operational definitions were defined by the research team which included an experienced information science researcher and experts in rehabilitation, BCI, and medical engineering. The methodology was based on the Cochrane handbook for systematic reviews of interventions and the PRISMA statement (Preferred Reporting Items for Systematic Reviews and Meta-Analyses) (Higgins and Green, 2011) and used the PICOS acronym to guide the search strategy development. In line with best practice, screening for inclusion at the abstract and the manuscript stages and during data extraction of the included studies was conducted independently by two researchers. Where disagreements arose, they were discussed among the reviewers first and then with an independent third party until a consensus was achieved.

The inclusion criteria for the review population were adults (>18 years) with confirmed diagnosis of stroke and at any stage of stroke recovery. No limitation by stroke etiology, first or recurrent event, or symptom presentation were applied. Adults with other known neurological diseases (e.g., spinal cord injury and multiple sclerosis) were excluded.

The interventions included in the review, broadly termed as “robotic gait training,” must comprise exoskeleton or other electromechanically assisted gait devices and be implemented in conjunction with biosignal (EEG and/or EMG) data capture as part of the study. For the purpose of this review, robotic devices could be either end-effector (electromechanically driven footplates simulating the phases of gait) or exoskeleton (robot-driven orthosis) gait devices.

Comparator populations were not a mandatory inclusion criterion, but studies that include a control group or a matched comparator group were considered eligible for inclusion. RCTs, cross-over, or quasi-randomized control studies, case–control studies, cohort studies, cross-sectional studies, case series, and case reports were all eligible for inclusion. Reviews, opinion pieces, editorials, and conference abstracts were excluded. This review was not designed to specifically examine the efficacy of the robotic gait interventions on stroke outcomes; rather, we were interested in the neural biosignals of EEG and or EMG themselves when recorded during robotic gait training after a stroke and how these signals interface with the robotic device.

### Information Sources

**Figure 1:**
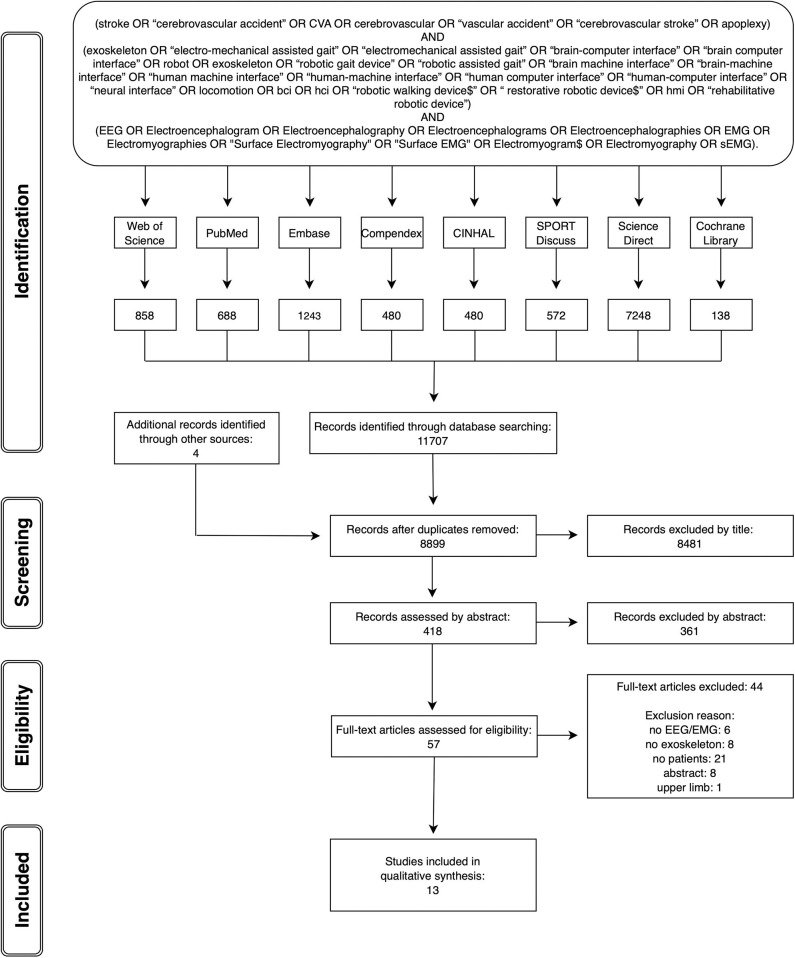
PRISMA flow chart with sample search strategy.

A systematic search and review of the literature was completed, which was compliant with the PRISMA guidelines (Moher et al., 2010). The following databases were searched from inception up to the 30th of November 2018: PubMed (1949–2018), EMBASE (1947–2018), Web of Science (1945–2018), COMPENDEX (1967–2018), CINAHL (1982–2018), SPORTDiscus (1985–2018), ScienceDirect (1997–2018), and Cochrane Library (1974–2018). No language, publication year, or publication status limits were applied to the database searches. Each database was searched using a comprehensive search strategy which was devised in conjunction with a librarian, including controlled vocabulary terms specific to each database and employing Boolean operators AND and OR. Gray literature was searched for in the following websites: http://bnci-horizon-2020.eu/database/data-sets and OpenGrey. A sample search strategy is provided as part of Figure 1.

### Study Identification and Selection

The citations identified were subjected to the following review process. Duplicate records were removed. The remaining studies were then reviewed independently by two reviewers against the established eligibility criteria in three stages: screening by title, screening by abstract, and screening by full text. An inclusive approach was taken, whereby if it was not clear whether a study fulfilled the necessary criteria for inclusion, it progressed to the next more in-depth review stage.

### Methodological Quality of the Included Studies

The reviewers independently documented the methodological quality of the included studies using the Effective Public Health Practice Public tool (EPHPP) in conjunction with the EPHPP dictionary for standardization. The EPHPP tool has been established as a reliable and valid tool in health research and is suitable to use across a range of differing study methodologies (Thomas et al., 2004, 2008). The disparity in ratings was discussed until a final decision was agreed.

Eight different sections of study quality to be applied as appropriate to the study type were addressed: selection bias, study design, confounders, blinding, data collection methods, withdrawals and dropouts, intervention integrity, and analysis. The tool provides an overall rating of either strong, moderate, or weak quality for each study.

### Data Extraction, Synthesis, and Analysis

Data were extracted from the included studies using a pre-agreed, standardized data collection form. The data extracted included (1) the characteristics of stroke study participants (including number, age, stroke type, stroke severity, and ambulatory ability), (2) type of robotic gait devices employed, (3) neural biosignal/s captured, (4) protocol reported for signal capture and processing, and (5) purpose and use of biosignal capture. Discrepancies in extraction, mainly related to the criteria for reporting biosignal processing, were resolved through a group discussion until a consensus was reached.

Narrative and tabular syntheses of data were proposed due to the heterogeneity of the study methodologies included. An overview of the studies meeting the inclusion criteria is initially provided, summarizing across the studies the stroke patient profiles, robotic devices, neural biosignal/s captured during robotic gait training after a stroke, and the purpose of the signal capture.

A summary of current integration of EEG and/or EMG data during robotic walking is presented next, with the current state of the art in closing the BCI/HMI loop in robotic gait training after a stroke being delineated.

The protocols for EEG and EMG signal collection are reported in a tabular format, with a narrative summary identifying the hardware and the software utilized where reported, the number of channels/leads used, and the sites chosen for signal capture.

EEG and EMG signal processing, as employed in the included studies, are again reported in a tabular format, with a summary identifying the frequency of signal capture, filtering processes, and software and algorithms used.

## Results

### Overall Summary of Studies Identified

The database searches were completed by end of November 2018. Figure 1 provides the PRISMA flow chart of the studies identified through database searching and through each stage of the review process. From 8,899 articles identified by the search strategy, 13 full papers fitting the inclusion criteria were included.

Tables 1, 2, which report the EEG and the EMG studies, respectively, detail the characteristics of the stroke participants, the robotic gait devices deployed, and the purpose of the neural bio-signal recording.

**Table 1:** Electroencephalography (EEG)-based robotic studies (participants and purpose).

**References**	**Robotic device**	**Stroke patients**	**Mobility level**	**Outcome measures**	**Purpose of EEG recording**	**Feedback to robot (Y/N)**	**Real-time feedback (Y/N)**	**Adverse events (Y/N)**
Calabrò et al. (2018)	EKSO	*N* = 40 H-C 69.0 ± 4.0 yrs RGT 67.0 ± 6.0 yrs OGT Type: I Side: 8L + 12R	FAC <5 MRC <4	10 MWT, RMI, TUG, sEMG, CSE, SMI, FPEC	Identify the cortical activations induced by gait training	N	N	Y
Contreras-Vidal et al. (2018)	H2 and continuous-time Kalman decoder	*N* = 6 C 53.5 ± 12.5 yrs Type: 2 I; 2 He; 2 M Side: 2L + 4R	NS	BBS, FGA, 6 MWT, TUG, FM, BI (pre/post)	Decoding gait kinematics	N	N	NS
He et al. (2014)	X1	*N* = 1 US 51 yrs Type: NS Side: 1L	FM-LL 12/34 BBS 38/56 FGA 13/30	EEG decoding accuracies for kinematics and EMG	Feasibility of decoding joint kinematics and muscle activity patterns	N	N	NS

*Patient data reported: sample number (N), stroke classified as (A) acute, (SA) subacute, (C) chronic, (US) undefined stroke and (H) hospitalized; age in years (yrs); robot gait training (RGT) and overground gait training (OGT); stroke type classified in (I) ischemic stroke, (He) hemorrhagic, (M) mixed; L, affected side—left; R, affected side—right. Control subject sample (CTRL) is reported, if any. NS: not specified in the manuscript*.

*Outcomes and mobility: 10 MWT, Ten-Meters Walking Test; RMI, Rivermead Mobility Index; TUG, Timed Up and Go; sEMG, surface electromyography; CSE, Corticospinal Excitability; SMI, Sensory-Motor Integration; FPEC, Fronto-Parietal Effective Connectivity; BBS, Berg Balance Score; FGA, Functional Gait Assessment; 6MWT, Six-Minute Walking Test; FM, Fugl–Meyer assessment; BI, Barthel Index; FAC, Functional Ambulation Classification; MRC, Medical Research Council scale for muscle strength; FM-LL, Fugl–Meyer Lower Limb Scale*.

**Table 2:** Electromyography (EMG)-based robotic studies (participants and purpose).

**References**	**Robotic device**	**Stroke Patients**	**Disability level**	**Outcome measures**	**Purpose of EMG recording**	**Feedback to robotic (Y/N)**	**Real-time feedback (Y/N)**	**Adverse events (Y/N)**
Androwis et al. (2018)	EKSO GT (EXO)	*N* = 5 A (first event) 51.0 ± 17.0 yrs Type: NS Side: 2L + 3R	FIM 26 ± 4	FIM	Test a novel EMG analysis (Burst Duration Similarity Index) and assess the neuromuscular adaptations in lower extremities muscles	N	N	NS
Calabrò et al. (2018)	EKSO (EXO)	*N* = 40 H-C 69.0 ± 4.0 yrs RGT 67.0 ± 6.0 yrs OGT Type: I Side: 8L + 12R RGT 9L + 11R OGT	FAC <5 MRC <4	10 MWT, RMI, TUG, sEMG, CSE, SMI, FPEC	Quantify gait parameters and compare mean muscle activity pre/post robotic and standard therapy	N	N	Y
Chisari et al. (2015)	Lokomat (EXO)	*N* = 15 H 62.0 ± 10.0 yrs Type: 10 I, 5 He Side: NS	Ability to walk for a few meters	FMMS, BBS, 10 MWT, TUG, 6 MWT	Strength and motor unit firing rate of vastus medialis	N	N	NS
Coenen et al. (2012)	Lokomat (EXO)	*N* = 10 C 55.0 ± 11.0 yrs CTRL = 10 47 ± 12 yrs Type: 5 I, 5 He Side: 8L + 2R	FAC = 5	sEMG during gait cycle	Compare EMG amplitude in robotic walking, overground walking for stroke patients, and overground walking for control subjects	N	N	NS
Fan and Yin (2013)	Lower extremity exoskeleton with a standing bed frame (EXO, non-commercial)	*N* = 3 H (2 SA, 1 C) 50.7 ± 19.2 yrs CTRL = 3 25.3 ± 1.5 Type: NS Side: 2L + 1R	NS	Exoskeleton forces and angles, joint ROM and active flexion/extension force	To decode movement and predict human motion inattention	Y	Y	NS
Gandolfi et al. (2017)	First mover (EE)	*N* = 2 H-SA 74 yrs CTRL = 10 65.4 ± 6.1 yrs Type: I Side: L	FAC = 0 TCT <12	sEMG, MI, MRC, AS	Explore the training effects on lower limb function and timing of muscle activation onset and offset	N	N	N
Gandolla et al. (2018)	EKSO GT (EXO)	*N* = 13 (8 A, 5 C) 52 ± 14 yrs Type: 7 I, 6 He Side: 8L + 5R	Tibialis anterior MRC <4 MAS <2 at hip, knee, ankle	GM, sEMG during gait cycle	(1) Computational calibration procedure, (2) gait cycle reference	Y	N	NS
He et al. (2014)	X1 (NASA) (EXO)	*N* = 1, 51 yrs CTRL = 2 33.8 ± 0.1 yrs Type: NS Side: L	FM-LL 12/34 BBS 38/56 FGA 13/30	EEG decoding accuracies for kinematics and EMG	Assess muscle activation pattern	N	N	NS
Hesse et al. (2010)	G-EO-Systems (EE)	*N* = 6 SA <75 yrs Type: I Side: 3L + 3R	Independent walker (>20 m, >0.25 m/s) Stair climber (aids/hand rails allowed)	sEMG activation pattern during floor walking and stairs climbing; FAC, RMI, MI, BI	Compare lower limb muscle activation with and without the robot	N	N	NS
Ping et al. (2013)	NaTUre-gaits (EXO non-commercial)	*N* = 1 H-C, 73 yrs SCI=2, 32 and 67 yrs CTRL = 4, age NS Type: NS Side: L	Moderate level of assistance to walk	sEMG during gait cycle	Investigate the timing and intensity of activity in the lower limb muscles during the use of the system	N	N	NS
Sloot et al. (2018)	Exosuit (EE)	*N* = 8, age NS Type: NS Side: NS	Walkers (level of assistance: NS)	sEMG, walking speed, energy cost of walking	Maximum EMG values during push-off and swing during walking with and without EE	N	N	NS
Srivastava et al. (2016)	ALEX II (EXO)	*N* = 12 SA-C (6 RGT, 6 BWSTT) RGT: 62.7 ± 12.7 yrs BWSTT: 58.8 ± 9.0 yrs Type: NS Side: 4L + 2R RGT 3L + 3R BWSTT	Walkers (level of assistance: NS)	TUG, 6 MWT, FGA, FM (pre/post)	Compare muscle activation timing during the gait cycle in RGT and BWTSS	Y	N	NS

*Robotic devices: EXO, exoskeleton; EE, end-effector. Patient data are reported as: N, sample number; A, classified as acute stroke; SA, classified as subacute stroke; C, classified as chronic stroke; H, hospitalized; SCI, spinal cord injury; RGT, robot gait training; BWSTT, body-weight supported treadmill training; OGT, overground gait training; I, stroke type classified in ischemic stroke; He, stroke type classified in hemorrhagic stroke; M, stroke type classified in mixed; L, affected side—left; R, affected side—right; CTRL, control subject sample is reported, if any; NS, not specified. Outcomes and mobility: FIM, Functional Independence Measure; FAC, Functional Ambulation Classification; MRC, Medical Research Council muscle strength; TCT, Trunk Control Test; MAS, Modified Ashworth Scale; FM-LL, Fugl–Meyer Lower Limb Scale; BBS, Berg Balance Score; FGA, Functional Gait Assessment; 10 MWT, Ten-Meter Walk Test; RMI, Rivermead Mobility Index; TUG, Timed Up and Go; sEMG, surface EMG; CSE, corticospinal excitability; SMI, sensory–motor Integration; FPEC, fronto-parietal effective connectivity; FMMS, Fugl–Meyer Motor Scale; 6 MWT, Six-Minute Walk Test; MI, Motricity Index; AS, Ashworth Scale for spasticity; GM, Gait Motor Index; BI, Barthel Index; FM, Fugl–Meyer assessment*.

A total of 96 out of the 122 individuals with stroke who were recruited in the studies received robot-assisted gait training on at least one occasion. Calabrò et al. recruited the largest cohort (40 stroke subjects, 20 of whom underwent robotic training) (Calabrò et al., 2018), whereas others reported a case study (Ping et al., 2013). The stroke participants differed widely across studies in terms of age profile, stroke type, stroke lateralization, and disability levels. Where reported, the ages ranged from 29 to 81 years of age. The laterality of the stroke event was described for 99 of the 122 stroke participants, 46 of whom had a right-sided stroke (with left hemiplegia). Two studies did not report stroke laterality (Chisari et al., 2015; Sloot et al., 2018). Stroke etiology was reported in 92 cases: 72 of which were ischemic in origin, 18 were hemorrhagic, and two were ischemic/hemorrhagic. Six studies did not provide information related to stroke type (Ping et al., 2013; He et al., 2014; Androwis et al., 2018; Sloot et al., 2018). The time from stroke to study participation was reported for 98 patients, with the majority (*N* = 57) recruited in the chronic phase of stroke. Three studies, comprising 13 subjects in total, selected stroke participants during the acute/subacute phase (Hesse et al., 2010; Gandolfi et al., 2017; Androwis et al., 2018). Three studies involved the collection of data from both chronic and acute/subacute phases of stroke (*N* = 10 in acute phase; *N* = 15 in chronic phase) (Fan and Yin, 2013; Srivastava et al., 2016; Gandolla et al., 2018). The stage of stroke recovery was not specified for the remaining 24 participants.

As noted in Tables 1, 2, a variety of methods were employed to describe the walking ability of the participants and, where comparable, the disability levels of the stroke study participants varied. Three authors adopted the Functional Ambulation Classification (FAC) as a standardized scale to describe the dependence levels in walking. Coenen et al. included fully independent walkers (FAC 5) (Coenen et al., 2012), Gandolfi et al. selected people who were unable to walk (FAC 0), (Gandolfi et al., 2017) and Calabrò et al. focused on stroke patients with gait impairment (FAC ≤ 4) (Calabrò et al., 2018). Three studies identified the participants as “walkers” but did not specify the level of assistance required, if any (Chisari et al., 2015; Srivastava et al., 2016; Sloot et al., 2018). Other studies described the participants' mean motor subscale score of the Functional Independence Measure (Androwis et al., 2018) [26 ± 4; where 13–38 indicate low scores for motor independence as guided by Itaya et al. (2017)], the Fugl–Meyer Lower Limb Scale (He et al., 2014) [12/34; where a cutoff score of <21 indicates lower mobility levels, as guided by Kwong and Ng (2019)], or strength of the lower limb tibialis anterior muscles of <4 on the MRC scale (Gandolla et al., 2018) or specified the level of assistance required to walk (Hesse et al., 2010; Ping et al., 2013). Two studies did not address the participants' walking status (Fan and Yin, 2013; Contreras-Vidal et al., 2018); however, one of these studies used the 6 MWT as a baseline score.

Exoskeleton devices were the most frequent robotic gait devices deployed in the studies included (*n* = 10 studies). The Lokomat (Lokomat® Hocoma, Switzerland) was used in two studies, EKSO (Ekso bionics®, USA) was used in three studies (two EKSO GT and one non-specified EKSO); the X1 (NASA, USA), the H2 (Technaid, Spain), the ALEX II (ROAR Laboratory, USA), and NaTUre-gaits (Nanyang Technological University, Singapore) devices were used in one study each. Fan and Yin combined a non-commercial lower extremity exoskeleton robot with a standing bed frame (Fan and Yin, 2013). Three end-effector devices were reported in the included studies [First Mover (Reha Technology AG, Switzerland), G-EO-Systems (Reha Technology AG, Switzerland), and Exosuit (Wyss Institute for Biologically Inspired Engineering, Harvard University, USA)].

### Closing the Loop Between Human and Robotic Device

No studies included in this review closed the loop in real time using EEG biosignals during robotic walking after a stroke, indicating that this field has not sufficiently evolved in a patient population such as stroke. One study described a multisensor, real-time movement prediction model that included sEMG of knee flexor and extensor muscles, joint angle, and force to determine the rehabilitation mode and the parameter settings in a bespoke exoskeleton (Fan and Yin, 2013). Errors in movement prediction were evident however during active training, when flexion and extension altered rapidly.

### EEG-Based Studies

Three of the thirteen studies included in this review recorded and analyzed EEG activity. As summarized in Table 1, two studies used EEG during robotic gait to decode gait kinematics (He et al., 2014; Contreras-Vidal et al., 2018) and muscle activity during walking (He et al., 2014). One study used EEG to determine frontoparietal connectivity as an outcome measure of neuroplasticity following a robotic gait training intervention (Calabrò et al., 2018).

Table 3 summarizes the EEG signal processing methods employed by the researchers. Contreras-Vidal et al. identified neural representation at the brain level for robotic gait using a powered H2 exoskeleton. A wireless, 64-channel, active electrode EEG-based system (BrainAmpDC, Brain Products, Inc., Munich), with continuous-time Kalman decoder operating on delta band, was utilized in five chronic stroke patients to demonstrate the feasibility of an EEG-based BCI-controlled rehabilitative robotic exoskeleton. The classification accuracy for predicting joint angles during gait was noted to improve with multiple training sessions and gait speed (Contreras-Vidal et al., 2018). The pilot study conducted by He et al., using a 10th-order unscented Kalman filter, demonstrated similar moderately high online decoding accuracies for joint kinematics during robotic gait but not for muscle activity patterns during robotic gait training in two healthy participants and one stroke survivor (He et al., 2014) using a multimodal interface comprising EEG [64-channel EEG (actiCap system, Brain Products GmbH, Munich, Germany)], EMG, and motion (goniometers), instrumented in conjunction with the X1 exoskeleton employed during 5-min overground walking sessions of three conditions: no robot, robot off (X1 in passive mode), and robot on (X1 in active mode). The final EEG-based study, an RCT by Calabrò et al. (*N* = 40 sub-acute and chronic stroke patients), employed 21-channel EEG as a measure of neuroplasticity using frontoparietal effective connectivity (FPEC) but did not interface with the robotic device directly. EEG was recorded using a high-input impedance amplifier (referential input noise <0.5 μVrms at 1÷20,000 Hz) of Brain Quick SystemPLUS (Micromed; Mogliano Veneto, Italy), wired to an EEG cap equipped with 21 Ag tin disk electrodes positioned according to the international 10–20 system. An electrooculogram (EOG) was also recorded for blinking artifact detection. EEG and EOG were sampled at 512 Hz, filtered at 0.3–70 Hz, and referenced to linked earlobes. The cortical activations induced by gait training from the EEG recordings were identified by using low-resolution brain electromagnetic tomography (LORETAKEY alpha-software). Structural equation modeling technique (or path analysis) was employed to measure the effective connectivity among the cortical activations induced by gait training. Improved FPEC was observed when robot-assisted gait training was included in the rehabilitation in comparison to conventional rehabilitation alone (*r* = 0.601, *p* < 0.001).

**Table 3:** Electroencephalography (EEG) signal processing in included studies.

**References**	**Protocol and analysis**	**Channels**	**Frequencies**	**Filtering**	**Reference**	**Additional**
Calabrò et al. (2018)	High-input impedance amplifier (Brain Quick SystemPLUS, IT) Eyes open recording pre-post rehab session Low-resolution brain electromagnetic tomography to identify cortical activations induced by gait training Structural equation modeling	21 (10–20 config)	512 Hz	BP, 0.3–70 Hz	Referenced to linked earlobes	Electrooculogram
Contreras-Vidal et al. (2018)	Wireless, active electrode EEG (BrainAmpDC, DE) Signals acquired during overground gait session Peripheral channels removed Detrend the remaining channels Down-sample to 100 Hz to match the frequency of H2 EEG and kinematics segmented in walk/stop epochs Principal component analysis applied to EEG data matrix to reduce the dimensionality 10th-order unscented Kalman filter to decode joint kinematics	64	1,000 Hz	Butterworth 4th-order Zero-phase BP, 0.1–3 Hz	FCz	Kinematic data acquired by H2
He et al. (2014)	actiCap system (Brain Products GmbH, DE) Data collected during robotic gait Peripheral channels removed Principal component analysis applied to the EEG data matrix to reduce the dimensionality Common average filter (CAR) 10th-order unscented Kalman filter to predict goniometer and electromyography measurements	64 (10–20 config)	1,000 Hz	BP, 0.01–100 Hz	FCz	EMG Biaxial electrogoniometer Hip and Knee angles measured by the X1

*Filter type: BP, band-pass; LP, low-pass; HP, high-pass*.

### EMG-Based Studies

Table 4 summarizes the EMG measurements from 12 studies included in this review. Only eight of the 12 studies defined the EMG device used: two studies used a Noraxon, two a BTS, one a DataLog, one a Motion Lab, and one a Porti system; one study used a self-made signal acquisition processor. Among these, five were wireless EMG devices.

**Table 4:** Electromyography (EMG) signal processing in included studies.

**References**	**Recording device and processing**	**Muscles**	**Hip**	**Knee**	**Ankle**	**Wireless**	**Frequencies**	**Filtering**	**Additional devices**
Androwis et al. (2018)	Noraxon (AZ, USA) Amplitude analysis: integrated EMG Timing analysis: Burst Duration Similarity Index	TA, SOL, RF, VL, BF, gastrocnemius	N	Y	Y	Y	2,520 Hz	Butterworth 4th-order Zero-lag BP, 20–300 Hz Notch, 60 Hz	Retroreflective markers
Calabrò et al. (2018)	8-ch BTS (IT) Root mean square for muscle activation	TA, SOL, RF, BF	N	Y	Y	Y	1,000 Hz	BP, 5–300 Hz	Accelerometer
Chisari et al. (2015)	Noraxon, Telemyo 2400T V2 SENIAM guidelines Reference electrode on the patella Frequencies at 50, 75, and 95% of the total power spectral density were estimated Root mean square normalized for the RMS of the maximum voluntary contraction	VM	N	Y	N	Y	3,000 Hz	Zero-lagBP, 20–500 Hz	Isokinetic dynamometer
Coenen et al. (2012)	16-ch Porti (NL) Envelope calculation: rectified EMG, 4th-order LP Butterworth 5 Hz	GM, TA, ST, RF, AL, GLM, GLm	Y	Y	Y	N	1,000 Hz	Butterworth 4th-order, HP, 20 Hz	Video gait analysis
Fan and Yin (2013)	2-ch self-made sEMG acquisition processor	BF and quadriceps	N	Y	N	NS	NS	BP, 10–500 Hz Notch, 50 Hz	Force sensors, angular encoders
Gandolfi et al. (2017)	Device not defined SENIAM guidelines Envelope representation	TA, RF, BF, gastrocnemius	N	Y	Y	NS	1,000 Hz	LP, 480 Hz	Pressure sensor (overshoes)
Gandolla et al. (2018)	FREEEMG (BTS Bioengineering, IT) SENIAM guidelines No processing (activation timing only)	TA, SOL, RF, ST	N	Y	Y	Y	NS	Butterworth 6th-order, HP, 20 Hz	
He et al. (2014)	8-ch DataLOG MWX8 (Biometrics)	TA, VL, BF, gastrocnemius	N	Y	Y	Y	1,000 Hz	BP, 20–460 Hz	Biaxial electrogoniometers, hip and knee angles measured by the X1
Hesse et al. (2010)	Device not defined SENIAM guidelines EMG mean onset and offset points of activation determined by thresholding the envelope	TA, VM, VL, RF, BF, GLm, gastrocnemius	Y	Y	Y	NS	1,000 Hz	1^st^-order LP, 500 Hz	Overshoe force sensors
Ping et al. (2013)	Device not defined EMG activity was acquired during the robotic gait and referred to the % of the gait cycle. Patients' EMG was confronted with the activity of a healthy control Research focuses on the shape of EMG profile, times of peak, or onset/cessation of myoelectric activity	TA, GM, VL, RF, ST, SM	N	Y	Y	NS	NS	NS	
Sloot et al. (2018)	EMG device not defined Compared maximum EMG values during push-off and swing between with and without robotic device	TA, GM, SOL	N	N	Y	NS	NS	NS	
Srivastava et al. (2016)	16-ch MA-416-003 Motion Lab System (LA) Signal normalization to its peak amplitude Non-negative matrix factorization (dimensionality reduction) to compute muscle modes and understand the effects of gait training on coordination	BF, VL, VM, RF, GLm, SOL, GL, GM, TA, medial hamstrings	Y	Y	Y	N	1,200 Hz	HP, 20 Hz	

*Muscle abbreviations: TA, tibialis anterior; GL, gastrocnemius lateralis; GM, gastrocnemius medialis; SOL, soleus; RF, rectus femoris; VL, vastus lateralis; VM, vastus medialis; BF, biceps femoris; ST, semitendinosus; SM, semimembranosus; AL, adductor longus; GLM, gluteus maximus; GLm, gluteus medius (GLm). Filter type: BP, band-pass; LP, low-pass; HP, high-pass*.

The majority of the studies collected EMG data to assess neuromuscular adaptations during robotic gait in stroke (Coenen et al., 2012; Ping et al., 2013; Chisari et al., 2015; Srivastava et al., 2016; Androwis et al., 2018; Calabrò et al., 2018; Sloot et al., 2018) or as an outcome measure following robotic training (Hesse et al., 2010; Chisari et al., 2015; Gandolfi et al., 2017). One study employed EMG activity as a calibration tool to identify individualized, optimal robotic parameters based on the gait index score derived from a normalized dataset (Gandolla et al., 2018). Every study used a symmetrical scheme for electrode placement, collecting EMG signals from both stroke-affected and contralateral sides, with the exception of one study (Srivastava et al., 2016) that collected EMG data from the paretic leg only. The number of muscle groups for EMG signal capture varied from only one muscle site (Quadriceps) (Chisari et al., 2015) to up to seven different muscle groups per limb (Hesse et al., 2010; Coenen et al., 2012), with no clear rationale for the muscle groups provided. Three studies referenced the guidelines used to identify optimal electrode placement (SENIAM guidelines) (Chisari et al., 2015; Gandolfi et al., 2017; Gandolla et al., 2018). Eleven out of the 13 studies tested the dorsi-flexors and the plantar-flexors of the ankle joint. Knee joint muscles were assessed by 12 studies. One of these studies recorded rectus femoris only (Chisari et al., 2015), whereas the others registered both flexor and extensor muscle groups. The hip musculature was addressed in three studies (Hesse et al., 2010; Coenen et al., 2012; Srivastava et al., 2016).

Muscle activity and timing of onset were registered and interpreted in relation to the gait cycle in 10 studies (Hesse et al., 2010; Coenen et al., 2012; Ping et al., 2013; Srivastava et al., 2016; Gandolfi et al., 2017; Androwis et al., 2018; Calabrò et al., 2018; Gandolla et al., 2018; Sloot et al., 2018). A variety of methods were employed, including instrumented gait analysis systems (Androwis et al., 2018), synchronized video analysis (Coenen et al., 2012; Ping et al., 2013), accelerometry (Calabrò et al., 2018), shoe-mounted sensors (Hesse et al., 2010; Gandolfi et al., 2017), or through the monophasic soleus muscle EMG activity and deactivation during gait, where the signal portion between two soleus muscle deactivations corresponds to a step cycle (Gandolla et al., 2018). The detailed protocols, where provided by the authors, are summarized in Table 5. Where explicitly reported, all studies set the EMG sampling frequency at or over 1,000 Hz in accordance with the Nyquist sampling principle. This was not specified in four studies (Fan and Yin, 2013; Ping et al., 2013; Gandolla et al., 2018; Sloot et al., 2018). Impedance was checked and kept below 5 kΩ by two studies (Hesse et al., 2010; Gandolfi et al., 2017), while the other studies did not specify impedance checking. The studies applied different signal filtering methods (Butterworth, high/low/band-pass filtering, keeping signals usually between 5/20–300/400/500 Hz). The signals were full-wave-rectified, and root mean square was applied to calculate the EMG amplitude and to provide a global overview of the muscle activity.

**Table 5:** Quality rating of included studies.

**References**	**Selection bias**	**Study design**	**Confounders**	**Blinding**	**Data collection methods**	**Withdrawal and dropouts**	**Total score**
Androwis et al. (2018)	Moderate	Moderate	N/A	N/A	Moderate	Moderate	Strong
Calabrò et al. (2018)	Moderate	Strong	Strong	Moderate	Strong	Strong	Strong
Chisari et al. (2015)	Moderate	Moderate	N/A	N/A	Moderate	Weak	Moderate
Coenen et al. (2012)	Moderate	Moderate	Weak	N/A	Weak	Weak	Weak
Contreras-Vidal et al. (2018)	Weak	Moderate	N/A	N/A	Strong	Strong	Moderate
Fan and Yin (2013)	Weak	Weak	Weak	N/A	Weak	Strong	Weak
Gandolfi et al. (2017)	Weak	Moderate	Weak	Moderate	Moderate	Strong	Weak
Gandolla et al. (2018)	Moderate	Weak	N/A	N/A	Strong	N/A	Moderate
He et al. (2014)	Weak	Weak	N/A	N/A	Moderate	N/A	Weak
Hesse et al. (2010)	Weak	Weak	N/A	N/A	Weak	Strong	Weak
Ping et al. (2013)	Weak	Moderate	Weak	N/A	Weak	N/A	Weak
Sloot et al. (2018)	Weak	Moderate	N/A	N/A	Weak	N/A	Weak
Srivastava et al. (2016)	Weak	Strong	Strong	Weak	Strong	Weak	Weak

*N/A, not applicable*.

### Co-registered EMG and EEG Data Collection

Only two papers (He et al., 2014; Calabrò et al., 2018) captured both EEG and EMG data. One study decoded the muscle activation patterns by scalp EEG signals during robotic walking, demonstrating reasonable success at decoding the hip and knee EMG activity in the affected leg of a stroke survivor (He et al., 2014). The authors cited difficulty with the EMG data collection, however, as the exoskeleton device and its attachments, in many cases, were located at the same anatomical sites as the EMG electrodes. The second study reported EMG and EEG as separate measures and was therefore not considered a co-registration of neural signals (Calabrò et al., 2018).

### Quality Review

As identified in Table 5, many studies were rated as “weak,” using the EPHPP guidance tool, primarily due to a potential selection bias during participant recruitment, thereby limiting their representation of the stroke population. Here the majority of studies failed to identify their recruitment strategy. Similarly, the studies received a lower quality rating where the reliability and the validity of the data collection methods were not explicitly reported.

## Discussion

This systematic review compiled the current state of the art in the use of neural biosignals during robotic gait training after a stroke. No studies that used EEG signals to close the loop between human and robotic gait device were identified. Two BCI studies that show promise (with adequate training) were identified for the classification of gait in an exoskeleton after a stroke with a view toward a future BCI application (He et al., 2014; Contreras-Vidal et al., 2018). The work presented by Contreras-Vidal (Contreras-Vidal et al., 2018) builds on a previously published framework proposed by this study group (Contreras-Vidal and Grossman, 2013). Otherwise, as with the majority of the EMG studies identified, the EEG signals were used as an outcome measure to evaluate RGT devices in stroke rehabilitation, for example, as an index of fronto-parietal connectivity to quantify neuroplastic changes (Calabrò et al., 2018). A recent systematic review of BCI rehabilitation in stroke supports this finding, where EEG was used to trigger neuromuscular electrical stimulation in the lower limb but not robotic gait devices to date (Cervera et al., 2018).

Specifying a search strategy that must include individuals with stroke in this systematic review yielded very limited EEG data. While this is informative with respect to the current state of the art in this area in stroke rehabilitation, it does not reflect the broader field of EEG-based control for robotic gait devices well. A recent systematic review by Al-Quraishi et al. (2018) comprehensively reported on EEG-based control for upper and lower limb exoskeletons and prostheses. In this review, 14 studies that used EEG-based control for lower limb movement, primarily in healthy subjects and individuals with spinal cord injury, were identified. Among those, nine studies targeted robotic gait-assistive devices (alone or in conjunction with an avatar), three used motor-imagery-only protocols with event-related desynchronization/resynchronization (ERD) (Do et al., 2013; Gordleeva et al., 2017; Lee et al., 2017), four used a movement-based protocol—with the EEG signal analysis undocumented in one (He et al., 2018b), and in the remaining three as ERD (Garcia-Cossio et al., 2015), combined ERD and movement-related cortical potential (MRCP) (López-Larraz et al., 2016), and exogenous steady-state visually evoked potentials with the visual stimuli representing robotic commands (Kwak et al., 2015). Two studies identified used a combination of motor imagery and movement using sensorimotor rhythms and MRCP (Liu et al., 2017) and event-related spectral pertubations (Donati et al., 2016). Notably, in one patient with a spinal cord injury, EEG signals were used to detect gait initiation to trigger the exoskeleton movement (López-Larraz et al., 2016). In another study with healthy individuals, online control of an overground exoskeleton using ERD in sensorimotor networks to train a classifier to identify two different mental states of walking forward intention or turning were demonstrated. In one body-weight-supported exoskeleton system, the user's intention was classified into active and passive walking phases using 62-channel EEG and power spectrum analysis in 8–30 Hz, normalized to the baseline condition to calculate ERD (Garcia-Cossio et al., 2015). The classification accuracies for active and passive walking with baseline were 94 and 93%, respectively, demonstrating the capability of BCI-assisted robotic training. The majority of EEG-based control in lower limb studies (*N* = 11; 79%) included in this cited review (Al-Quraishi et al., 2018) were markedly published from 2015 onwards, indicating a relatively new research area and, in part, explaining the poor penetration in the stroke population identified in this current systematic review. Another review of brain–machine interfaces for controlling lower limb powered robotic systems (He et al., 2018a) identifies that the most common studies in this area are classification-based studies of walk vs. stand tasks in healthy subjects and system performance is not clearly presented in these studies. Several challenges were summarized in this review, including EEG denoising, safety, and responsiveness. Furthermore, it concluded that suitable performance metrics and more clinical trials were required to advance research and development in the field.

One study that investigated closed-loop control between human and robotic gait device involving three stroke survivors was identified in this systematic review (Fan and Yin, 2013). EMG activity levels from knee flexor and extensor muscle groups were measured and a multisystem decoding paradigm, which included EMG in addition to joint angle and force production data, allowed the robotic command to be altered. High error rates in the commands generated during active movement were observed when flexion and extension activity alternated rapidly, limiting application in the clinical setting (Fan and Yin, 2013). EMG methods for motor intent identification have previously been noted to have significant limitations in stroke in that they are only appropriate for patients who can produce some voluntary movement or high-enough levels of muscle activity and are not suitable for individuals with severe motor impairment, profound muscle fatigue, or abnormally coactivated muscles (Li et al., 2018). Concerns have also been raised that continuous EMG control may indeed reinforce pathological movement rather than encouraging the recovery of normal motion patterns (Krebs et al., 2003).

EMG has been combined with EEG to improve the recognition of movement intent in the upper limb (Bhagat et al., 2016) in the BCI literature. The current review identified two studies that recorded EEG and EMG. However, the two neural biosignals were not used in conjunction in either study to decode movement. One study reported these measures separately (Calabrò et al., 2018), while the other decoded EMG activity in the lower limbs using EEG during robotic walking in one stroke subject (He et al., 2014).

One example was identified in the literature where the best power transfer between subject and robot was achieved through a fine-tuning procedure for robotic parameters based on optimized EMG activity during the gait cycle (Gandolla et al., 2018); in this context, sEMG could prove to be a useful tool to optimize the patient–robot interaction in the clinical setting. However, the current lack of personalization of robotic gait command derived from neural biosignals and the limited ability to tailor robotic training to participant effort and to rehabilitative goals aligned with motor (re)learning principles limit their capacity as truly restorative devices in stroke rehabilitation. Robotic gait devices and the technological advancements enabling their continued development have been the preserve of the field of engineering (Pons, 2010). Translational research that examines deployment of devices in a clinical population must now also draw from expertise in rehabilitation and clinical research. This paper includes input from experts in the field of neurology, rehabilitation, bioengineering, and BCI engineering, discusses shortcomings in the papers identified, and makes recommendations to advance this field of research. A quick reference guide DESIRED (Table 6) has been developed by the group to identify a minimum reporting data set as a standard for future studies and the rationale and evidence base guiding these recommendations are described in detail next.

**Table 6:** The DESIRED checklist: minimal reporting dataset for neural biosignals during robotic gait after a stroke.

**DESIRED**	**Minimal requirement**	**Recommended but not mandatory**
Description of study methodology	Adequate description of the clinical study type: e.g., randomized controlled trial; observational study: case study; case series, cross-sectional, pre–post design; mixed methods	Published guideline for study type referenced and checklist completed
Explicit reporting of stroke participant recruitment strategy	Recruitment method stated. Focus of the study on acute/subacute/chronic phases of stroke stated. Number of potential participants approached and number who entered the study described	Participants' location is described, e.g., in-patient acute or rehabilitation center; out-patient rehabilitation center; community dwelling and attending community services or no current rehabilitation provided at the time of recruitment
Stroke participant profile	Stroke pathology, e.g., ischemic or hemorrhagic, stroke side (at brain level); time from stroke to study participation; provide an index of gait impairment, e.g., functional ambulatory category; identify the presence and the type of sensory impairment where relevant	Stroke severity described, e.g., National Institutes of Health Stroke Scale (score included); cognitive level/s described
Intervention described using FITT principles	Frequency, intensity, time, and type of intervention reported	Report who delivered the intervention; the level of skill and training of the interventionist and whether there was fidelity of interventionist
Robotic gait training	Device and manufacturer; exoskeleton vs. end-effector device; over-ground vs. treadmill walking	Robotic mode settings described, e.g., whether step trajectory is fully supported by the robotic device or whether the device allows participant contribution to the step generated
Electroencephalography data capture	At minimum 32-electrode EEG with inclusion of activity from the central pre-motor/motor/sensorimotor and posterior parietal cortical areas to categorize walking from rest and ensuring frequency bands in 8–12 Hz (alpha/mu), 12–28 Hz (beta), and 28–40 Hz (low gamma) are represented in the data collection For motion trajectory prediction, global analysis to identify the most suitable features (potential or band-power time-series, low-delta, mu, or beta frequency band and all cortical areas) currently recommended	State if active electrodes are used and, if so, the planned data filtering. Use of source-resolved EEG dynamics during walking (mobile brain/body imaging) Minimization of artifactual contamination of lateral electrode signals by neck muscle electromyography during walking by blind source separation (typically by independent component analysis or frequency clustering) Potential time-series of the low-delta EEG oscillations or band-power time-series of the mu and beta EEG oscillations may hold the most information for motion trajectory prediction but further supporting research is required
Electromyography data capture	Minimum of two agonist/antagonist paired muscles in the distal and proximal compartment of stroke-affected and contralateral leg. Tibialis anterior, soleus, rectus femoris, and vastus lateralis recommended where stroke impairment and robotic gait device allow clean signal to be collected. Identification of the minimal crosstalk area of the muscle for electrode placement using the guidelines given by Basmajian and Blummenstein, updated by Blanc and Dimanico, with the axis of the electrodes directed parallel to the muscle fibers	Sensor placement checks for single muscles by checking for crosstalk on the other collected traces is recommended where spasticity or muscle shortening is present. Power spectral density computation may be useful when using exoskeleton gait devices to unravel unwanted electrical interference from electrical actuators, battery packs, and cables

The majority of papers identified reported methodologies related to the devices, biosignals, and/or model development as appropriate to the domain of engineering. As a consequence, when considered as clinical studies in a stroke population and assessed using a broadly applicable quality rating tool (Thomas et al., 2004), the majority of studies were deemed to have a weak methodology. Consistent problems identified across studies related to the selection of stroke subjects and to the reporting of the validity and the reliability of the outcome measures employed. Guidelines with quality control checklists are available across a range of clinical study methodologies, for example, RCTs (Campbell et al., 2012), observational studies (Von Elm et al., 2014), and qualitative methodologies (Booth et al., 2014). When introducing participants with stroke or other neurological pathologies to robotic and/or neural signal-based studies, it is recommended that the authors familiarize themselves with the criteria expected based on the study type to be reported in the paper.

It is interesting to note that none of the papers reviewed provided a rationale for their selection of the stroke participants, and limited details on stroke pathology, stroke laterality, and stroke severity levels were documented. The time from stroke, for example, is something that further warrants attention. After focal damage, there is a brief, approximately 3 months, window of heightened plasticity, the so-called opportunity window which, in combination with training protocols, leads to large gains in motor function (Zeiler and Krakauer, 2013). Emerging evidence now supports smaller, plastic, and non-compensatory recovery in the chronic stages after a stroke also (Mrachacz-Kersting et al., 2015; Carvalho et al., 2018). To compare the neural biosignals and their utility in robotic gait training after a stroke across studies and to allow the results to be interpreted correctly, it is imperative to report this information. No consensus was observed across studies with respect to the gait impairment level of those included in the studies and ranged from those fully and independently mobile to those who are wheelchair dependent, again limiting the conclusions that can be drawn across studies. To stratify the findings from future studies, a minimum data set for participants with stroke is recommended and summarized as: stroke type, laterality, time from stroke to inclusion in the study, and functional ambulatory category (Mehrholz et al., 2007). The impairment of sensation also needs to be taken into account, given that accurate motor control can only be exerted with correct sensory and proprioceptive input. An index of stroke severity would also be a useful addition, for example, the National Institutes of Health Stroke Scale score (Ortiz and Sacco, 2014), as well as the level of cognitive function of the participants, if this is not a stated inclusion/exclusion criterion. To best replicate clinical application, it is advised that only the participants with gait impairment are included in the research.

A review of the brain–machine interface for lower limb systems after a stroke, published in 2015, concluded that additional research and development were required to advance this field (Soekadar et al., 2015). This systematic review now identifies that EEG data use during robotic gait after a stroke remains sub-optimal to closing the loop between person and robot. It is acknowledged that EEG activity during walking is not well-understood in general and discordance exists in the literature on the temporal and the spectral patterns of cortical dynamics during walking (Wagner et al., 2012, 2014; Seeber et al., 2014, 2015; Bradford et al., 2015; Bruijn et al., 2015; Bulea et al., 2015; Storzer et al., 2016; Winslow et al., 2016; Artoni et al., 2017; Luu et al., 2017; Oliveira et al., 2017). A number of research groups (Bradford et al., 2015; Bruijn et al., 2015; Bulea et al., 2015; Winslow et al., 2016; Artoni et al., 2017; Luu et al., 2017; Oliveira et al., 2017) report event-related (de)synchronization (ERD/S) (i.e., an event-related power change) in 8–12 Hz (alpha/mu) and 12–28 Hz (beta) oscillations after the onset of stepping/walking task (Bradford et al., 2015; Bruijn et al., 2015; Bulea et al., 2015; Winslow et al., 2016; Artoni et al., 2017; Luu et al., 2017; Oliveira et al., 2017), while other research groups report ERS at 28–40 Hz (low gamma) during early and mid-swing and ERD toward the end of the swing phase and during double support (Wagner et al., 2012, 2014; Seeber et al., 2014, 2015; Storzer et al., 2016). The literature does call attention to the importance of the central pre-motor/motor/sensorimotor and posterior parietal cortical areas in neural signal generation during the walking tasks. Thus, for the separation of walking from rest periods, we recommend the above-described cortical areas and the frequency bands are represented in the data collected and processed.

Decoding the 3D motion trajectory of the lower limbs is a more challenging objective (Georgopoulos et al., 2005). In BCI, this method poses an ideal solution for controlling a robotic device as the applied signal processing algorithm reconstructs the track of the intended movement. To date, most joint trajectory decoding studies have focused on reconstructing the movement of the upper limbs (Bradberry et al., 2010) and fingers (Paek et al., 2014) using 0.5–2 Hz slow cortical potentials (SCP) or band-power time-series of mu and beta bands (Korik et al., 2015, 2016, 2018). Motion trajectory prediction has successfully been applied to lower limb kinematics during treadmill walking in healthy individuals by Presacco et al. (2011, 2012) using SCP time-series. Here topographical analysis did not identify a significant pattern of lower limb movement-related cortical areas. Two studies included in this current review identified the utility of the 0.1–3 Hz frequency band for decoding kinematic data (He et al., 2014; Contreras-Vidal et al., 2018) and EMG kinetics during robotic walking after a stroke (He et al., 2014). One of these studies primarily focused on the frontal, temporal, and parietal brain regions (He et al., 2014), while others removed the peripheral channels susceptible to facial/cranial movement-related noise (Contreras-Vidal et al., 2018). Thus, as decoding the motion of lower limbs during walking is a nascent area, we still recommend a global analysis to identify the most suitable features (potential or band-power time-series, low-delta, mu, or beta frequency band and all cortical areas). However, the SCP/mu and beta band-power time-series extracted from the central motor and posterior parietal areas most likely contain maximal information for decoding lower limb movement trajectories.

From the present review, the lack of a standardized EMG recording protocol when applied to people with stroke-related disability in interaction with exoskeletons is evident. This limitation hampers the constitution of shared database repositories and pooling of data. A protocol and reported methodology should include a minimum dataset of muscles, dimensions, and positioning criteria of the surface EMG electrodes, interelectrode distance, techniques to verify the system selectivity, and technical sampling requirements (e.g., amplifier characteristics, electrode diameter, and impedance limits). The guidelines also suggest the inclusion of additional details relating to signal analysis pipelines, such as filtering and signal quality checks (Blanc and Dimanico, 2010; Merletti and Farina, 2016; Benedetti et al., 2017).

Currently, no consensus exists on targeting specific muscle groups during gait analysis in stroke survivors. The surface EMG of agonist and antagonist lower limb muscle activity during gait is emerging as an effective way of defining motor control during spontaneous movement in stroke (Srivastava et al., 2019). A minimum set of agonist and antagonist muscles in the distal and the proximal compartment of the leg needs to be defined to provide comprehensive muscle recruitment patterns and muscle synergies during the gait cycle after a stroke, which could be helpful for future HMI. Additionally, for individuals after a stroke, sEMG data from both legs (stroke affected and non-affected sides) should be collected as motor deficits are not only associated with the stroke-affected side but also of the non-affected side during spontaneous walking (Parvataneni et al., 2007; Bagnato et al., 2009; Tseng and Morton, 2010; Raja et al., 2012). Recent reviews of muscle synergies in post-stroke gait and robotic gait devices support the need for better standardization of muscles chosen for EMG data capture (Molteni et al., 2018; Van Criekinge et al., 2019). Tibialis anterior, soleus, gastrocnemius, and rectus femoris were noted to be most commonly assessed in all muscle synergy studies after a stroke (Van Criekinge et al., 2019). Considering best clinical practice and the need to record agonist and antagonist muscles during gait, a minimum representative muscle set to be targeted in future studies is recommended as bilateral: tibialis anterior, soleus, rectus femoris, and vastus lateralis, where possible. Alternate muscle/s selection may need to be defined by the participant's stroke-related muscle impairment/s or the robotic gait device and its positioning at specific anatomical landmarks for sensor placement, leading to muscle group exclusion.

Only three studies included in this review referred to a guideline document used for the correct positioning of electrodes on muscles. Failure to do this limits the reliable recording of muscle signals and does not address the challenge of avoiding “crosstalk” (diffused signal components coming from co-active or inactive adjacent muscles) (Basmajian, 1983). Correct sensor positioning aims to minimize this phenomenon and allows the researchers to identify a real co-contraction of agonist and antagonist muscle groups, which is common after a stroke. Basmajian and Blummenstein provide instructions to identify the minimal crosstalk area (MCA) for electrode placement on superficial muscles during gait (Basmajian and Blumenstein, 1980; Basmajian, 1983; Campanini et al., 2007; Blanc and Dimanico, 2010). Although the Surface EMG for Non-invasive Assessment of Muscles (SENIAM) guidelines referenced in the included studies are readily available and easy to use (www.seniam.org; Merletti, 2000), the MCA locations defined by Basmajian and Blummenstein, subsequently updated by Blanc and Dimanico (Blanc and Dimanico, 2010), have been proven to be superior to the SENIAM guidelines (Campanini et al., 2007). MCA identification would now be a minimum standard recommendation to follow in this field when studying the EMG timing during gait (Campanini et al., 2007), with the axis of the electrodes directed parallel to the muscle fibers to increase selectivity (Blanc and Dimanico, 2010). Additional quality assurance measures during robotic gait after a stroke, where feasible with the constraints of the device itself, could include sensor placement checks—performed by eliciting contractions of a single muscle and checking for crosstalk on the other collected traces (Benedetti et al., 2017) and strongly recommended where spasticity or muscle shortening may alter placement accuracy—and data check to ascertain the shape of the power spectral density (PSD) of the signal to ensure meaningful content (Merlo and Campanini, 2010), free of movement artifacts. When recording surface EMG in sessions that include the use of exoskeletons, electrical interference on EMG signals coming from electrical actuators, battery packs, and cables is not unexpected, and the PSD computation could prove to be a powerful tool to unravel such unwanted events.

## Limitations

The authors acknowledge that while no language limits were applied when searching across databases, no papers were returned in languages other than English. As such, it is possible that additional manuscripts exist that were not identified through this search strategy. The search also returned studies with heterogeneous use of neural bio-signals, including as an outcome measurement only. While these were included in the review, their purpose was not in line with the primary focus of this review. However, in unifying all studies in this area irrespective of their set purpose, biosignal collection and interpretation in this field could be generalized and commented on constructively.

## Conclusion

Overall, while there are ever-growing technological advances in robotics, actuators, and sensors, advances in applications to entrain robotic commands with biosignals for gait training in clinical populations such as stroke have been considerably slower. EEG recording in stroke, where the pathology is at the brain level, has been problematic when compared to other neurological pathologies such as spinal cord injury (Castermans et al., 2014), and similarly EMG recording on the stroke-affected side can be problematic (Sarasola-Sanz et al., 2017; Li et al., 2018). Uncertainty still exists in the literature on the best choice of EEG metric (Goh et al., 2018) and in the ability of EMG to respond accurately in real time (Fan and Yin, 2013). This review, summarizing the current state of the art in neural interface during robotic-assisted gait training after a stroke, identifies a lack of standardization in data collection in this field and provides guidance for study design and reporting future studies. Promising findings for decoding movement during robotic gait after a stroke and potential for EMG, in conjunction with other measurement modes to close the loop, have been elucidated.

## Data Availability Statement

All datasets generated for this study are included in the article/supplementary material.

## Author Contributions

OL, AD, EG, FM, CG, and DC developed the concept for this systematic review. OL, MT, RO, and CF contributed to the development of the search strategy, review of papers, data extraction, and synthesis sections. All the authors had equal involvement in the drafting and the revising of the review article by unique contribution to the intellectual content and consensus among all the authors on the content.

## Conflict of Interest

CG and RO are employed at g.tec Medical Engineering GmbH, Austria. The remaining authors declare that the research was conducted in the absence of any commercial or financial relationships that could be construed as a potential conflict of interest.
